# Time course of effective connectivity associated with perspective taking in utterance comprehension

**DOI:** 10.3389/fnhum.2023.1179230

**Published:** 2023-11-06

**Authors:** Shingo Tokimoto, Naoko Tokimoto

**Affiliations:** ^1^Department of English Language Studies, Mejiro University, Tokyo, Japan; ^2^Department of Performing Arts, Shobi University, Saitama, Japan

**Keywords:** perspective taking, effective connectivity, speech comprehension, electroencephalogram, source localization, partial directed coherence, individual difference in sociality

## Abstract

This study discusses the effective connectivity in the brain and its time course in realizing perspective taking in verbal communication through electroencephalogram (EEG) associated with the understanding of Japanese utterances. We manipulated perspective taking in a sentence with the Japanese subsidiary verbs *-ageru* and *-kureru*, which mean “to give”. We measured the EEG during the auditory presentation of the sentences with a multichannel electroencephalograph, and the partial directed coherence and its temporal variations were analyzed using the source localization method to examine causal interactions between nineteen regions of interest in the brain. Three different processing stages were recognized on the basis of the connectivity hubs, direction of information flow, increase or decrease in flow, and temporal variation. We suggest that perspective taking in speech comprehension is realized by interactions between the mentalizing network, mirror neuron network, and executive control network. Furthermore, we found that individual differences in the sociality of typically developing adult speakers were systematically related to effective connectivity. In particular, attention switching was deeply concerned with perspective taking in real time, and the precuneus played a crucial role in implementing individual differences.

## 1. Introduction: perspective taking in verbal communication

We can generally assume that the main purpose of verbal communication is to communicate intention. However, understanding the literal meaning of an utterance is often insufficient for verbal communication because the speaker's intention is often implicit. For example, speaker A's utterance in (1) can be understood as an indirect question in which the speaker asks where they can refuel by saying that they do not have gasoline.

(1) A is standing by an obviously immobilized car and is approached by B.
A: I am out of petrol.
B: There is a garage around the corner (Grice, 1975).

The implied intention in (1) is generally called *conversational implicature*. Although the details of the mechanism for understanding conversational implicature are still unclear, we can assume that the implicature is derived by pragmatic inference from the literal meaning of an utterance and its context. It is generally believed that the literal meaning of an utterance is constructed compositionally from the meaning and order of the words and morphemes and that word meanings and syntactic rules are shared within a language community. However, the referents of some linguistic forms cannot be determined independently of their context. A typical example is personal pronouns, where the first-person pronoun *I* and second-person pronoun *you* denote the speaker and the listener, respectively. However, since speakers change frequently in a conversation, *I* and *you* must be understood as the interlocutor and the interpreter, respectively, when the interlocutor is the speaker. Therefore, the listener, as the interpreter, must take the speaker's perspective to understand *I* and *you* correctly, which means that perspective taking frequently occurs during conversations.

Another example of perspective taking in English is the contrast between indirect and direct narratives, as shown in (2). The propositional meanings of (2-a, b) are identical. However, *I* in (2-b) refers to *my father*, and therefore, the speaker must take the perspective of their father within the quotation marks.

(2) a. My father told me yesterday that he would go fishing today.
b. My father said to me yesterday, “I will go fishing tomorrow.”

Many linguistic forms involve perspective taking, but the default perspective is assumed to be the speaker's own. One typical construction that involves a perspective shift is a passive sentence. For example, in (3-a, b), the situations described by the two sentences are identical, but the perspectives are different. The active sentence in (3-a) is neutral about the perspective, whereas the passive sentence in (3-b) describes the situation from a perspective closer to Bill than to John.

(3) a. John respects Bill.
b. Bill is respected by John.

If *John* and *Bill* are brothers, they can be expressed as *Bill's brother* or *John's brother*, respectively. However, in this case, while (4-a) is a natural expression, (4-b) sounds awkward. This is explained by the fact that there are two different perspectives in (4-b), with the passive sentence showing the perspective of the subject *John's brother* (Bill), while *John's brother* describes Bill from John's perspective. On the other hand, the perspective in (4-a) is consistent because the passive construction and expression *his brother* (Bill's brother) both indicate the perspective of Bill.

(4) a. Bill is respected by his brother.
b. #John's brother is respected by him.

This constraint is proposed in Kuno (1987) as a constraint on the perspective in verbal communication, and the perspective is referred to as *linguistic empathy* in (5).

(5) a. Ban on conflicting linguistic empathy fociA single sentence cannot contain logical conflicts in empathy relationships (Kuno, 1987, p.207).
b. Linguistic empathyLinguistic empathy refers to the speaker's identification with a person or thing that participates in an event or state that is described in a sentence, which may vary in degree (Kuno, 1987, p. 206).

Various linguistic forms are associated with linguistic empathy, but the psychological/neural mechanism of perspective taking in real-time verbal communication is unknown. Many animals communicate with voice, but no animals other than humans vocalize (signs) or understand another individual's voice (sign) from the perspective of that individual. In this respect, perspective taking is an ability unique to humans.

In this study, we examined the neural substrate of the unconscious and automatic mechanism of perspective taking in real-time utterance comprehension through electroencephalogram (EEG) measurements. Recent neuroscience studies have suggested that brain function can be viewed through flexible and integrated processing mechanisms, and cognitive function is generally understood to be implemented by whole-brain connectivity (Anderson and Barbey, 2023). The brain network for perspective taking has been discussed primarily by examining the neural substrate in autism spectrum disorder (ASD). Three recent functional magnetic resonance imaging (fMRI) studies on perspective taking deeply relevant to the present study are briefly reviewed below.

Mizuno et al. (2011) was an early study that used language as material and discussed brain connectivity for typically developing (TD) individuals and individuals with ASD. Mizuno et al. (2011) presented the participants with two kinds of images in an MRI scanner, namely, images in which the interlocutor uttered *I* and *you* and images in which the interlocutor and the participant were referred to by their proper names. The former was viewed as the perspective-shift condition because the perspective shifted to the interlocutor, while the latter was viewed as the control condition. The degree of blood oxygen level-dependent signal synchronization between the precuneus and right anterior insula was compared between the perspective-shift and control conditions using fMRI imaging. The functional connectivity associated with perspective taking was evaluated by the correlation between the signal intensity in the two regions. The correlation was significantly greater in the perspective-shift condition than in the control condition. The experiments were conducted on the basis of the theoretical background that ASD is due to reduced frontal-occipital connectivity (Just et al., 2004; Kana et al., 2006, 2009), and the correlation was weaker for ASD speakers than for TD speakers, as expected.

Komeda et al. (2015) compared the brain functional connectivity for self- and other-reference processing between TD and ASD participants, accounting for the personality traits of ASD speakers. Komeda et al. (2015) presented TD and ASD participants with sentences describing a person's personality in an MRI scanner and asked them to judge whether the sentences applied to them. The descriptions were manipulated in two ways: characteristics that were associated with ASD and whether the target of the descriptions was the participant or others. For example, *I would rather be alone than with others. Do you agree with the sentence?* was presented for a self-judgment on a characteristic associated with ASD, and *Yuya would rather be with others than alone. Do you think you are similar to him?* was presented for a judgment on another person with a characteristic associated with TD individuals (Yuya is a typical Japanese male name). Komeda et al. (2015) specified the ventromedial prefrontal cortex (vmPFC) as the seed region and examined the functional connectivity between the vmPFC and other brain areas. In comparison with participants with ASD, TD participants showed greater functional connectivity for others' judgment than for self-judgment during nonautistic character judgments between the vmPFC and left inferior frontal gyrus (IFG), left precentral gyrus, right dorsolateral prefrontal cortex (dlPFC), left superior temporal gyrus (STG), dorsomedial prefrontal cortex (dmPFC), and right middle frontal gyrus (MFG). Komeda et al. (2015) claimed that empathic responses in TD individuals were produced by collaboration between frontal and posterior areas, whereas empathic responses in individuals with ASD were produced by collaboration within frontal areas.

Hashimoto et al. (2016) conducted an fMRI experiment in which the participants performed self- and other-evaluation tasks by taking either their own or another person's perspective. The experimental stimuli were Japanese sentences in the form of *A think(s) that B is/are C*, where A and B refer to either *you* (self) or *your mother* (other) and C represents an adjective describing a personality trait. For example, *You/your mother think(s) that you/your mother are/is cheerful*. The participant was asked to read the sentence and decide whether they agreed with it by pressing a button. Hashimoto et al. (2016) specified the left sensorimotor cortex (SMC) as the seed for functional connectivity analyses because this region showed the largest activation effect for different perspectives and because it is a major node in the mirror neuron system (Pineda, 2008). Hashimoto et al. (2016) observed increased connectivity between the left SMC and the right middle cingulate cortex (MCC)/precuneus and left posterior cingulate cortex (PCC)/precuneus when the participants took the perspective of others. According to Hashimoto et al. (2016), no significant difference in brain activation was observed between the ASD and TD groups when taking the perspective of others, whereas a significant main effect of perspective taking was observed in terms of alterations in functional connectivity in the ASD group.

Although the seed location was different in Komeda et al. (2015) and Hashimoto et al. (2016), the regions associated with perspective taking were similar, that is, the language areas (Broca's area and Wernicke's area), the mentalizing network, the mirror neuron network, and the executive control network. These studies are rigorous in their manipulation of perspective taking; however, their experimental tasks do not directly reflect the process of perspective taking in verbal communication. Since perspective taking frequently occurs in real-time verbal communication, the present study examines the effective connectivity associated with perspective taking in real-time speech comprehension by manipulating the perspective shift in an utterance and analyzing the associated EEG. Although EEG analyses of brain connectivity have reduced spatial resolution than fMRI analyses, EEG methods have several advantages: (1) temporal variations in connectivity can be analyzed (Michel and He, 2019), (2) different frequency bands can be analyzed, and (3) causal relationships can be evaluated.

In addition, previous studies on brain connectivity during perspective taking have mainly focused on the neural substrate of individuals with ASD compared to TD individuals, and it remains unclear whether individual differences in connectivity can be observed for TD individuals. With the growing neuroscientific interest in human social cognition, the correlation between the behavioral and physiological indices of participants and their individual differences in sociality has been widely discussed in experimental settings. Regarding language processing, Fairchild and Papafragou (2021) demonstrated that individual differences in theory of mind were associated with pragmatic competence in neurotypical adults across different pragmatic phenomena, that is, understanding of scalar implicature, indirect utterances, and metaphors. Perspective taking will not require inference to derive non-literal meanings or contextual implications, but it is fundamental in pragmatic processing in daily verbal communication. Thus, it is possible that individual differences in sociality correlate with the behavioral and physiological indices associated with perspective taking. The autism-spectrum quotient (AQ) (Baron-Cohen et al., 2001) has been widely used as a measure of individual differences in sociality. The AQ was originally designed to measure variation in autistic traits in nonclinical samples. Autistic traits are common in the general population (Skuse et al., 2005; Hoekstra et al., 2008; Ronald and Hoekstra, 2011), and patients with ASD are widely recognized as individuals at the extreme end of a normal distribution of autistic traits (Constantino and Todd, 2003). Some researchers have reported significant correlations between the social behavior of healthy individuals and their AQ (sub)scales [e.g., face viewing (Wegner-Clemens et al., 2020); social functioning (pro-social activity, interpersonal communication, independence-performance, independence-competence, recreation, and social engagement) (Demizu et al., 2022)]. The AQ subscales of communication and imagination, which can be relevant in understanding the speaker's intention, are expected to correlate with behavioral and physiological indices of language understanding in general, and the subscale of social skill can be a manifestation of individual tendencies in the application of linguistic rules. Furthermore, the subscales of attention switching and local details can be related to the individual differences in perspective shift in a sentence discussed in this study below.

Regarding the effect of sex, studies in nonhuman animals and younger human populations suggest that sex differences in empathy have phylogenetic and ontogenetic roots in biology and that they are not merely cultural byproducts driven by socialization (Christov-Moore et al., 2014). It is widely known that disorders of impaired empathy, including autism, are more common in males than females (Baron-Cohen, 2002; Baron-Cohen et al., 2005). We can also find many studies on neurotypical adults suggesting that females are generally more empathic and altruistic than males. For example, females are faster and more accurate than males in recognizing facial expressions (Hampson et al., 2006) and in recognizing bodily emotions, such as identifying actions as happier, sadder, angrier, or no different from a neutral action (Alaerts et al., 2011). Greenberg et al. (2023), on the other hand, analyzed the results of the Reading the Mind in the Eyes Test (Baron-Cohen et al., 2001) as a test of theory of mind performed in fifty-seven countries (including Japan) and demonstrated higher average performance on the test among females. Furthermore, eye tracking research by Schmid et al. (2011) demonstrated that females tended to process facial expressions more globally (i.e., attending to the whole face rather than local areas) than males and were more accurate in emotion recognition. For the sex differences in EEG, Brink et al. (2012) recorded the EEG associated with semantic (in)congruency, such as *You wash your hands with soap/horse and water*, and with (in)congruency of voice-based speaker identity, such as *I cannot sleep without my teddy bear in my arms* spoken by a six-year-old boy or an adult male speaker. Brink et al. (2012) observed a significant event-related negativity around the latency of 400 ms (N400) for semantic incongruency for both male and female participants, whereas they observed a significant N400 for incongruency of speaker identity only for female participants.

Regarding the effect of age, Greenberg et al. (2023) showed that the scores of the Reading the Mind in the Eyes Test increased after the age of 16, peaking at 20.25 years for females and 20.48 years for males and gradually decreasing thereafter.

Therefore, in the present study, individual differences in sociality were assessed by AQ in addition to age and sex, and their correlations with behavioral responses and brain connectivity were examined. The research questions of the present study are enumerated in (6).

(6) a. Does the effective connectivity associated with perspective taking change over time during speech processing?
b. Does the effective connectivity associated with perspective taking vary in different frequency bands?
c. Does the effective connectivity associated with perspective taking vary with sex, age, and individual differences in sociality among typically developing individuals?

## 2. Methods

### 2.1. Participants

Twenty-two native Japanese speakers, aged between 20 and 46 years (*M* = 29.32, *SD* = 9.30, 12 females), participated in this study. The participants had normal or corrected-to-normal vision and had no history of neurological/psychiatric disorders. All the participants were right-handed, as assessed by the handedness questionnaire (Oldfield, 1971). After signing the informed consent form, participants participated in the EEG measurement experiment and answered the AQ after the EEG measurement. The participants were paid. This study was approved by the Ethics Committee of Shobi University.^1^

### 2.2. Materials

We manipulated the presence or absence of a perspective shift during utterance comprehension utilizing the Japanese verbs *ageru* and *kureru*. The literal meaning of both *ageru* and *kureru* is “to give,” and these verbs can function as subsidiary benefactive verbs to form various compound verbs. For example, the propositional meanings of (7-a) and (8-a) are the same as those of (7-b) and (8-b), respectively. However, *-ageta* (gave) in (7-b) indicates that the speaker is describing the event from the perspective of the subject, *watashi* (I), whereas *-kureta* (gave) in (8-b) indicates that the speaker is describing the event from the perspective of the dative object, *watashi*, where *-nom, -dat*, and *-acc* denote the nominative, dative, and accusative cases, respectively. The subsidiary verbs and the corresponding foci of the perspectives in (7-b) and (8-b) are underlined.

(7)     a.    Watashi-ga Maruyama-san-ni            bentoo-o
    I-nom         Mr./Ms. Maruyama-dat   lunch box-acc
    katta.
    bought
    b.    Watashi-ga Maruyama-san-ni            bentoo-o
    I-nom          Mr./Ms. Maruyama-dat    lunch box-acc
    katte-ageta.
    buy-gave (to him/her)
    “I bought a lunch box for Mr./Ms. Maruyama.”

(8)     a.    Nakajima-san-ga            watashi-ni   bentoo-o
    Mr./Ms. Nakajima-nom I-dat             lunch box-acc
    katta.
    bought
    b.     Nakajima-san-ga           watashi-ni   bentoo-o
    Mr./Ms. Nakajima-nom I-dat             lunch box-acc
    katte-kureta.
    buy-gave (to me)
    “Mr./Ms. Nakajima bought a lunch box for me.”

Japanese speakers can take the perspective of a person with whom they have a mentally close relationship in a sentence. In (9), for example, the speaker takes the perspective of *ane* (the speaker's elder sister) in (9-a) and that of *otooto* (the speaker's younger brother) in (9-b). This is because *-ageta* requires the perspective of the subject, whereas *-kureta* requires that of the dative object.

(9)      a.    Ane-ga     Maruyama-san-ni            bentoo-o
    sister-nom Mr./Ms. Maruyama-dat   lunch box-acc
    katte-ageta.
    buy-gave (to him/her)
    “My sister bought a lunch box for Mr./Ms. Maruyama.”
      b.    Nakajima-san-ga       otooto-ni          bentoo-o
    Mr./Ms. Nakajima-nom brother-dat lunch box-acc
    katte-kureta.
    buy-gave (to him)
    “Mr./Ms. Nakajima bought a lunch box for my
    brother.”

As briefly discussed in the introduction section, Mizuno et al. (2011) used the first- and second-person pronouns (*I* and *you*) to manipulate perspective-taking. That is, the first-person pronoun refers to the speaker, but it refers to the interlocutor when the interlocutor is the speaker. Therefore, when the interlocutor is the speaker, the comprehender must take the perspective of the interlocutor to identify the referent of the pronoun. In the same way, the second-person pronoun refers to the interlocutor, but it refers to the comprehender when the interlocutor is the speaker. The comprehender therefore must take the perspective of the interlocutor to identify the referent of *you*. The use of first- and second-person pronouns shows that the perspective frequently alternates between speakers in conversation. We therefore assume here that the comprehender takes the perspective of the interlocutor by default. The perspective of the interlocutor is maintained in (7-b) and (8-b). On the other hand, the perspective shifts from the interlocutor to *ane* in (9-a) and to *otooto* in (9-b). In the present study, two types of sentences, (7-b) and (8-b), were combined into one condition with no perspective shift, and similarly, (9-a) and (9-b) were combined into one condition with a perspective shift. Accordingly, we created sixty no-perspective-shift sentences and sixty perspective-shift sentences, each of which included thirty sentences with *-ageru* and thirty with *-kureru*. In terms of temporal expression, Japanese verbs have two forms, one corresponding to past tense and the other corresponding to non-past tense. Half of the sixty sentences were in the past tense, and the remaining sentences were in the non-past tense. In total, two hundred forty experimental sentences were counterbalanced and divided into two stimulus sets, each including one hundred twenty sentences.

Forty control sentences were included in the main session, and they were anomalous due to the conflicting linguistic empathy in the sentence. In (10-a), for example, the sentence-final *-ageta* requires the perspective of the subject *Matsunaga-san* (Mr./Ms. Matsunaga), whereas the first-person pronoun *watashi* requires the perspective of the speaker, which is referred to as the speech act empathy hierarchy by Kuno (1987), as shown in (10-c).

(10)      a.    *Matsunaga-san-ga           watashi-ni    melon-o
         Mr./Ms. Matsunaga-nom I-dat            melon-acc
         kitte-ageta.
         cut-gave (to me)
         “Mr./Ms. Matsunaga cut me a melon for me.”
        b.     ?Musume-ga         Okumura-san-ni          coffee-o
    my daughter-nom Mr./Ms. Okumura-dat coffee-acc
        irete-kureta.
        make-gave (to him/her)
         “My daughter made Mr./Ms. Okumura a cup of coffee
         (for Mr./Ms. Okumura).”
        c.      Speech act empathy hierarchy
    The speaker cannot empathize with someone else more than with himself/herself (Kuno, 1987, p. 212).

The anomaly in (10-a) is thus explained by the ban on conflicting linguistic empathy foci in (5) because two different perspectives (linguistic empathy) are involved, namely, the perspective of *Matsunaga-san* required by *-ageta* and that of the speaker required by *watashi*. In (10-b), on the other hand, *-kureta* requires the perspective of the dative object *Okumura-san* (Mr./Ms. Okumura-dat), and the subject *musume* (my daughter) is psychologically close to the speaker (her parent); thus, it is easier for the speaker to take the perspective of the subject (*musume*). Therefore, to appropriately understand the situation described by (10-b), the listener must retrieve the context in which the speaker is (temporarily) psychologically closer to Mr./Ms. Okumura than his/her daughter. The example (10-b) is thus likely judged as anomalous because the retrieval of the appropriate context to understand the situation in (10-b) is costly.

The stimulus sentences were synthesized into the voices of three women to control their prosody and vocal emotions in their auditory presentation. Female voices were adopted because they were easier to recognize than male voices. The main session included one hundred twenty experimental sentences and forty control sentences, for a total of one hundred sixty sentences.

### 2.3. Procedure

**Figure 1:**
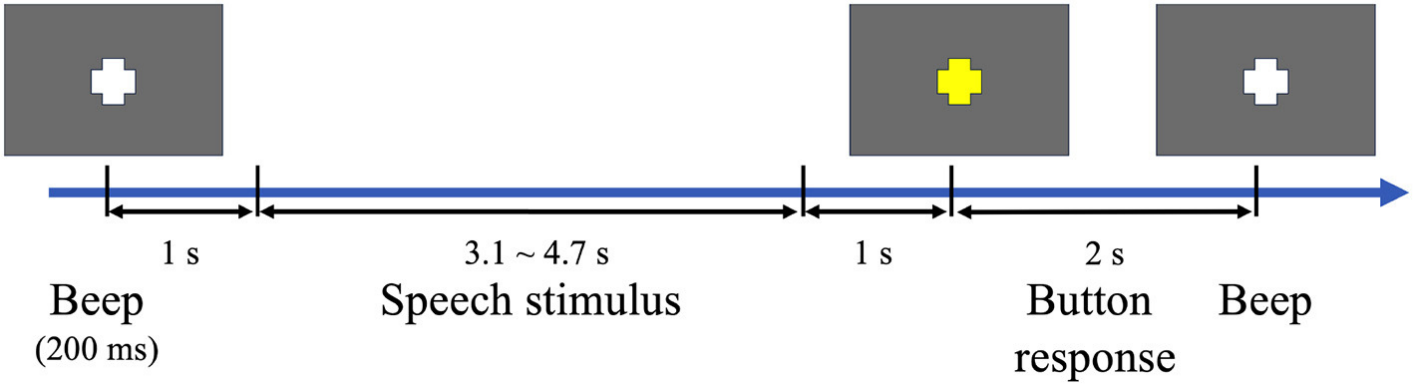
Sequence of stimuli and possible responses in a trial.

The participants were seated in an electrically and acoustically shielded EEG chamber 1 m in front of a 19-inch LCD monitor. A beep sound indicated the beginning of a trial, and a white fixation point was visually presented in the center of the display. A speech stimulus was auditorily presented 1 s after the beep. The fixation point turned yellow 1 s after the end of the speech stimulus, and the participants were asked to press a button if they judged the speech to be unnatural. The lengths of the speech stimuli ranged from 3.1 to 4.7 s, and thus, each trial lasted 7.1–8.7 s. Figure 1 shows the sequence of the experimental stimuli and the possible responses by the participants.

The order of the presentation of the stimulus sentences was randomized for each participant. The experiment was controlled using presentation software (Neurobehavioral Systems). The practice session consisted of ten trials. The main session consisted of three blocks, and the participants were allowed to rest for 3–5 min between the blocks. The experimental sessions, including the instruction and application of the electrodes, lasted ~1.5 h. The participants completed the AQ in Japanese (Wakabayashi et al., 2006) after their EEG recordings. The basic statistics of the participants are shown in Table 1. We found no significant difference between female and male participants for the seven considered indices.

**Table 1:** Basic statistics of the participants' responses to the Japanese version of the autism-spectrum quotient.

	**Age**	**AQ total**	**Communication**	**Social skill**	**Imagination**	**Attention switching**	**Local details**
**12 females**							
Mean	32.00	15.42	2.58	3.08	2.92	3.42	4.75
SD	10.17	8.38	2.94	2.31	1.24	2.75	2.09
Maximum	46	31	9	8	4	7	8
Minimum	20	4	0	0	1	0	1
**10 males**							
Mean	26.10	20.90	4.00	3.70	4.10	5.20	5.90
SD	7.37	9.42	1.83	2.54	2.28	1.55	2.96
Maximum	44	31	7	9	7	8	10
Minimum	20	4	2	0	1	3	2

### 2.4. EEG recording

The EEG signals were recorded using a 32-channel EEG amplifier (Brain Amp DC, Brain Products, Germany) with an active electrode recording system (actiCAP, Brain Products; extended 10–20 montage). The signals were sampled at 2.5 kHz, with a bandpass filter of 0.1 to 1,000 Hz applied and the reference electrode positioned at FCz. Vertical and horizontal electrooculograms (EOGs) were simultaneously recorded with electrodes placed below the right eye and at the outer canthus of the left eye. The electrode impedance was maintained at <20 kΩ during the sessions. The EEG data were continuously acquired using Brain Vision Recorder software (Brain Products). The average EEG recording time was 22.28 min (SD = 4.78 min).

### 2.5. EEG data preprocessing

The acquired EEG data were processed offline using EEGLAB (Delorme and Makeig, 2004). The following preprocessing steps were performed. (1) The data were high-pass filtered at 1 Hz to minimize low drifts with respect to the reference at FCz. (2) Line noise was removed using the CleanLine plugin in EEGLAB. (3) High-amplitude artifacts were removed from the EEG data using artifact subspace reconstruction (Mullen et al., 2015). (4) The data were decomposed using an adaptive mixture of independent component (IC) analyzers (AMICA) (Palmer et al., 2007). (5) The best-fitting single-equivalent current dipole was calculated for each IC to match the scalp projection of each IC source using a standardized three-shell boundary element head model. The electrode locations were aligned according to the 10–20 system with a standard brain model (Montreal Neurological Institute). (6) The ICLabel plugin in EEGLAB estimates the probabilities of the following sources for each IC: brain neural activity, EOG measurements, muscle potentials, electrocardiogram measurements, line noise, channel noise, and other sources. The classifier of ICLabel was trained on thousands of manually labeled ICs and hundreds of thousands of unlabeled ICs collected by the Swartz Center for Computational Neuroscience (Pion-Tonachini et al., 2017). We chose ICs for which the probability of brain neural activity was >70% for the following analyses. (7) ICs were excluded from further analyses if the equivalent dipole model explained <85% of the variance in the corresponding IC scalp map. The average number of rejected ICs for the twenty-two participants was 15.73 (SD = 2.31); thus, the average number of remaining ICs was 26.27. (8) The data were segmented into time epochs from −2 to 3 s relative to the event markers.

Several EEG and magnetoencephalographic experiments on perspective taking have focused on time-frequency spectra (Hari et al., 1998; Sakihara et al., 2012; Woodruff et al., 2016). Woodruff et al. (2016) presented participants with a series of photographs of happy, sad, angry, and neutral faces. The participants indicated how the emotion of the actor made them feel (self condition) and which of the four emotions was displayed in each photograph (other condition) in two experimental blocks. Woodruff et al. (2016) observed β suppression at the F3 and C3 electrodes in the other condition and β enhancement at the F4, Fz, C3, C4, and Cz electrodes in the self condition. If the neural substrates associated with perspective taking were common (in part) among different modalities, we could predict β suppression for perspective-shift utterances, in which the perspective shifted from the speaker to another person, in contrast to no-perspective-shift utterances, in which the default perspective of the speaker was maintained.

## 3. Results

### 3.1. Behavioral responses

The mean percentage of utterances that were judged to be “unnatural” was 9.8% (SD = 11.8%) for the perspective-shift condition, 5.3% (SD = 6.8%) for the no-perspective-shift condition, and 68.8% (SD = 16.6%) for the control condition. A one-factor ANOVA for the unnatural judgment rates with the three utterance types as within-subject factors indicated a significant effect of the utterance type [$F_{\left(2,\;42\right)}=247.0,MSe=0.469,p<0.001,\eta_{p}^{2}=0.92$]. The judgment rate for the control condition was significantly greater than that for the perspective-shift condition [*p* < 0.001 (Bonferroni correction)], and the judgment rate for the perspective-shift condition was significantly greater than that for the no-perspective-shift condition [*p* = 0.012 (Bonferroni correction)].

To examine the effect of individual sociality on the judgments, we calculated the correlation coefficients between the three judgment rates and the participants' subscales on the AQ. The judgment rate for the control condition was significantly negatively correlated with the social skill subscale in the AQ (*r* = −0.476, *p* = 0.025). The judgment rate for the no-perspective-shift condition was significantly negatively correlated with the age of the participants and significantly positively correlated with the attention switching subscale (*r* = 0.472, *p* = 0.027). The judgment rate for the perspective-shift condition was significantly negatively correlated with the age of the participants (*r* = −0.455, *p* = 0.033).

### 3.2. Event-related potential and event-related spectral power

**Figure 2:**
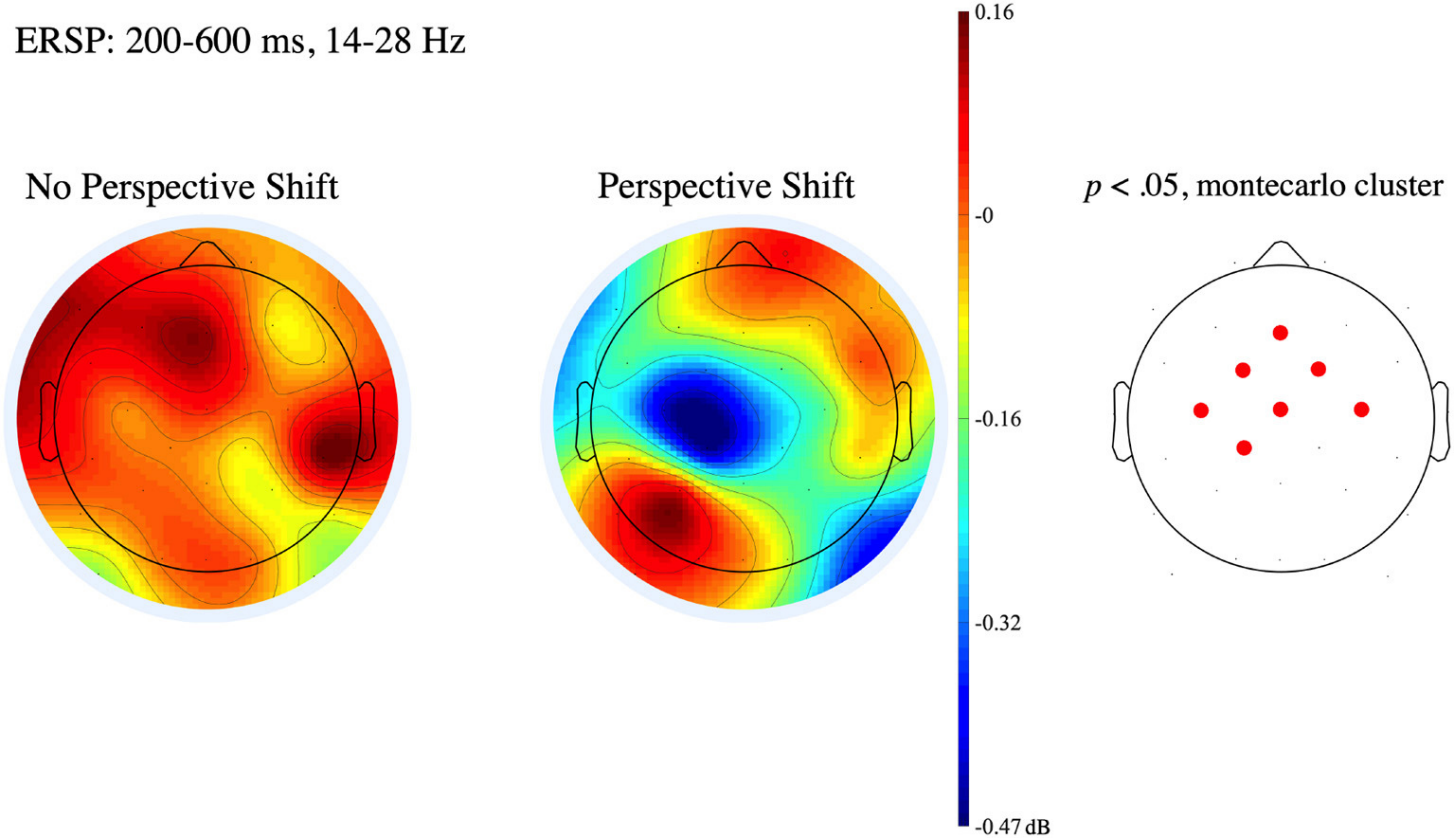
Mean topographies of the ERSPs time-locked to the onset of *ageru/ta* and *kureru/ta* in the β band (14–28 Hz) from 200 to 600 ms, with the poststimulus baseline set from 0 to 100 ms. The **left** side shows the no-perspective-shift condition, and the **center** shows the perspective-shift condition. The electrode sites at which significant differences were found using the cluster-based permutation test (*p* < 0.05) are shown in red in the right topography. The scalp color, corresponding to the color bar, represents the average increase or decrease in amplitude in the β band of the EEG from 200 to 600 ms after the input of *ageru/ta* and *kureru/ta* in the form of a ratio, with the average amplitude of 0–100 ms as 0 dB (Makeig, 1993). We observe a significant suppression in the β band at the centro-frontal electrodes in the perspective-shift condition.

To examine the neural effects of perspective shifting, the EEG data were analyzed with the no-perspective-shift and perspective-shift conditions as within-subject factors. The analyses of the effects of the conditions on the event-related potential (ERP) and event-related spectral power (ERSP) were performed using the STUDY command structure in EEGLAB. non-parametric random permutation statistics were computed to test the significance of the condition effects, and the comparison between the conditions was corrected by cluster-based permutation tests (Maris and Oostenveld, 2007). In the present study, we computed 2,000 random permutations and compared them to the *t* values for the mean condition differences. The ERPs and ERSPs in the θ (5–7 Hz), α (8–12 Hz), β (14–28 Hz), and γ (30–34 Hz) bands were calculated time-locked to the onsets of *-ageru/ta* and *-kureru/ta* for every 100 ms time window, and the baseline was set to 100 ms after their onset. We found no significant difference in the ERPs between the two conditions. However, We observed a significant β suppression for perspective-shift utterances compared with no-perspective-shift utterances in the frontocentral region. Figure 2 shows the mean topographies of the ERSPs in the β band in the latency from 200 to 600 ms.

### 3.3. Connectivity analyses

**Figure 3:**
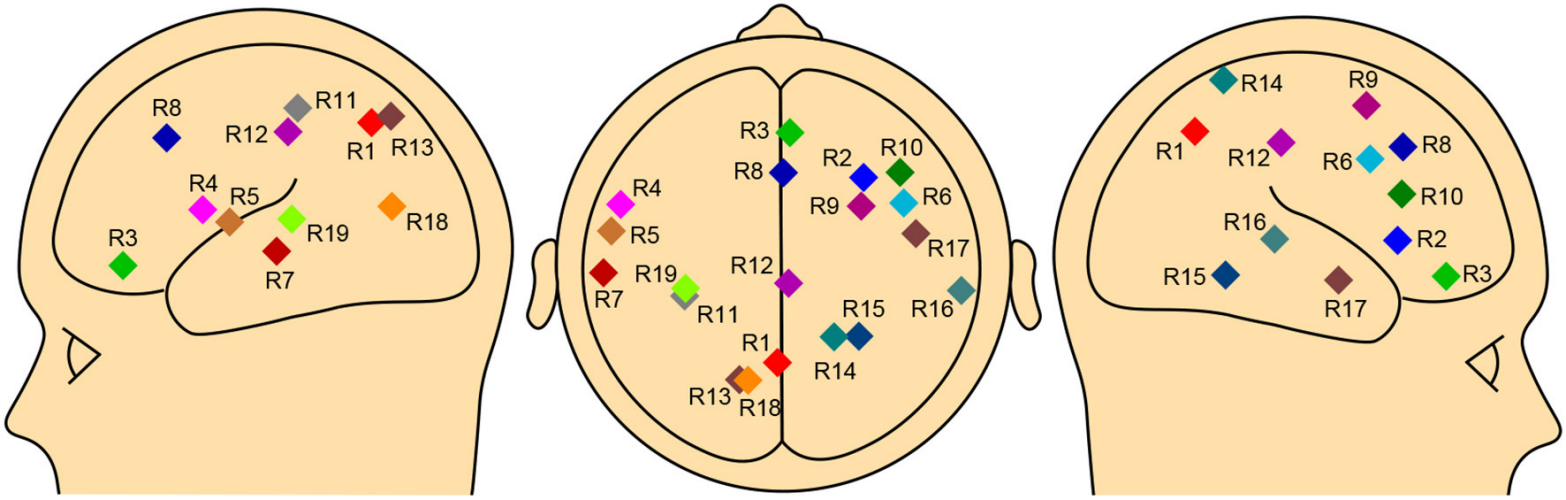
Nineteen regions of interest. **(Left)** Sagittal view of the left hemisphere, **(Center)** horizontal view, **(Right)** sagittal view of the right hemisphere.

We can find no preceding research on the effective connectivity for perspective taking in real-time language comprehension. In this study, therefore, we employed the nineteen regions of interest (ROIs) discussed in recent fMRI studies on perspective taking with language material briefly reviewed in the introduction section, namely, Mizuno et al. (2011), Komeda et al. (2015), and Hashimoto et al. (2016), to position the present study as a development of the previous studies and to enrich the theoretical interpretation of the results. Table 2 shows the hemisphere, structure, MNI coordinates, and Brodmann area of each ROI, and Figure 3 shows the locations of the nineteen ROIs. R1 and R2 were cited from Mizuno et al. (2011), R3 to R10 were from Komeda et al. (2015), and R11 to R19 were from Hashimoto et al. (2016), for which the main effect of perspective was greater for TD than for ASD speakers.^2^

**Table 2:** Nineteen regions of interest, including the hemisphere, structure, MNI coordinates, and Brodmann area (BA).

**Region**	**Hemisphere**	**Structure**	**MNI coordinates**			
			***x***	***y***	***z***	**BA**
R1		Precuneus	0	−64	50	7
R2	Right	Anterior insula	32	26	6	13
R3		vmPFC	4	48	−8	10
R4	Left	Inferior frontal gyrus	−60	12	16	44
R5	Left	Precentral gyrus	−64	0	12	6
R6	Right	dlPFC	48	14	38	9
R7	Left	Superior temporal gyrus	−68	−20	0	22
R8		dmPFC	2	28	44	8
R9	Right	Middle frontal gyrus	32	12	60	6
R10	Right	dlPFC	46	28	24	46
R11	Left	Sensorimotor cortex	−36	−30	56	3
R12	Right	MCC/precuneus	4	−26	46	31
R13	Left	PCC/precuneus	−14	−72	52	7
R14	Right	Superior parietal cortex	22	−52	70	7
R15	Right	Hippocampus gyrus	30	−50	−8	19
R16	Right	Superior temporal cortex	70	−28	6	42
R17	Right	Superior temporal cortex	52	0	−10	22
R18	Left	Middle occipital gyrus	−12	−72	16	18
R19	Left	Insula	−36	−28	12	13

vmPFC, ventromedial prefrontal cortex; dlPFC, dorsolateral prefrontal cortex; MCC, middle cingulate cortex; PCC, posterior cingulate cortex.

A widely known episode suggestive of the mental function of the vmPFC is the case of Phineas Gage (1823–1860), who was involved in an explosion during railroad construction in 1848. As a result of the explosion, an iron bar ~1 m long and weighing 6 kg penetrated his head. The accident left Gage blind in his left eye, but he lived on his own for eleven years and eight months after the accident, working at various jobs. Before the accident, Gage was a mentally stable and competent worker, but after the accident, his persona reportedly changed to be very capricious, irreverent, and haphazard. According to Damasio (1994) and Damasio et al. (1994), who analyzed photographs of Gage's skull preserved in a medical museum, the areas of Gage's brain that were damaged were the bilateral vmPFC, which caused impairment in rational decision making and emotional processing (however, according to Ratiu et al., 2004, Gage's brain damage was only to the medial and lateral orbitofrontal cortex and dlPFC in the left frontal lobe).

The neural responses to the “trolley problem” are also suggestive of the function of the vmPFC. The trolley problem is a thought experiment by Thomson (1976) that requires moral judgment in sacrificial dilemmas. In the “switch” and “footbridge” scenarios below, a utilitarian decision to sacrifice one person is required to save five lives. According to Awad et al. (2020), who examined sacrificial dilemmas among seventy thousand people in forty-two countries and in ten languages, the decision to save five lives at the expense of one is commonly more difficult cross-culturally in the footbridge scenario than in the switch scenario.

#### 3.3.1. Switch scenario

Edward is the driver of a trolley, whose brakes have just failed. On the track ahead of him are five people; the banks are so steep that they will not be able to get off the track in time. The track has a spur leading off to the right, and Edward can turn the trolley onto it. Unfortunately, there is one person on the right-hand track. Edward can turn the trolley, killing the one; or he can refrain from turning the trolley, killing the five (Thomson, 1976, p. 206).

#### 3.3.2. Footbridge scenario

George is on a footbridge over the trolley tracks. He knows trolleys and can see that the one approaching the bridge is out of control. On the track back of the bridge, there are five people; the banks are so steep that they will not be able to get off the track in time. George knows that the only way to stop an out-of-control trolley is to drop a very heavy weight into its path. However, the only available, sufficiently heavy weight is a fat man, also watching the trolley from the footbridge. George can shove the fat man onto the track in the path of the trolley, killing the fat man; or he can refrain from doing this, letting the five die (Thomson, 1976, p. 206–207).

Greene et al. (2001) and Greene et al. (2004) argue that the thought of pushing a person in front of a trolley elicits a strong negative emotion that drives moral disapproval. While utilitarian reasoning prevails in the absence of a strong negative emotion in the switch scenario, the strong negative emotion and utilitarian reasoning may conflict in the footbridge scenario. Emotional responses are assumed to be partially represented by the medial PFC, and cognitive control over the conflict between negative emotion, and utilitarian reasoning is assumed to be implemented by the dlPFC. In fact, Koenigs et al. (2007) reported that patients with bilateral focal damage to the vmPFC exhibited abnormally utilitarian judgment patterns when facing moral dilemmas.

Our EEG data were transformed from the electrode space into the source space with the montages for the nineteen ROIs. A source montage reconstructs approximate source waveforms. In the current study, the source waveforms were calculated following the principle of a generalized montage, giving weights to all thirty channels on the scalp. The transformation was based on the scalp topographies resulting from focal brain activities (e.g., from dipole and volume conductor modeling) and on the principles of linear algebra, resulting in an efficient spatial filter (Scherg et al., 2002, 2019; Michel and He, 2019). Some researchers have suggested that source-level EEG data reconstructed from scalp EEG data are reliable for measuring connectivity, whereas the interpretation of connectivity measures based on sensor-level EEG recordings is not straightforward (Moezzi and Goldsworthy, 2018). Furthermore, calculating the source-level connectivity based on sensor-level EEG data can eliminate the influence of volume conduction and field propagation (He et al., 2019).

To measure the directed information flow among the nineteen ROIs, we used BESA connectivity (BESA GmbH, version 1.0) to compute the partial directed coherence (PDC) in the frequency domain. The PDC is a multivariate directional connectivity measure that reflects the direct interrelations between signals (Baccalá and Sameshima, 2001). The magnitude of the PDC is defined as


$$\begin{array}{l} |{\text{PDC}}_{ij}\left(f\right)|=\frac{\left|\Lambda_{ij}\left(f\right)\right|}{\sqrt{\overset{N}{\underset{k=1}{\sum }}|\Lambda_{kj}\left(f\right){|}^{2}}}, \end{array}$$

where Λ_*ij*_(*f*) is an element of Λ(*f*) = H^−1^(*f*) (the inverse matrix of the transfer function) (BESA, 2019). PDC_*ij*_(*f*) describes the directional flow of information between the *j*th and *i*th signals (*j*→*i*). The PDC is normalized to take values between 0 and 1. Thus, the transmission ratio from signal *j* to signal *i* and the total outflow from signal *j* [the sum along the columns of Λ(*f*)] are obtained. The PDC is assumed to be computationally more efficient and more robust than the directed transfer function because it does not involve any matrix inversion (He et al., 2014; Cao et al., 2021). In the current study, the source-level EEG data were first transformed into the time–frequency domain using the complex demodulation method. Complex demodulation is a technique for describing the amplitude and phase of a given frequency component of a time series as functions of time, which provides a uniform frequency resolution across the analyzed bandwidth (Hao et al., 1992). The PDC was computed in the frequency domain by employing a non-parametric spectral factorization approach in the θ (5–7 Hz), α (8–12 Hz), β (14–28 Hz), and γ (30–40 Hz) bands.

#### 3.3.3. Effective connectivity between the nineteen ROIs

**Figure 4:**
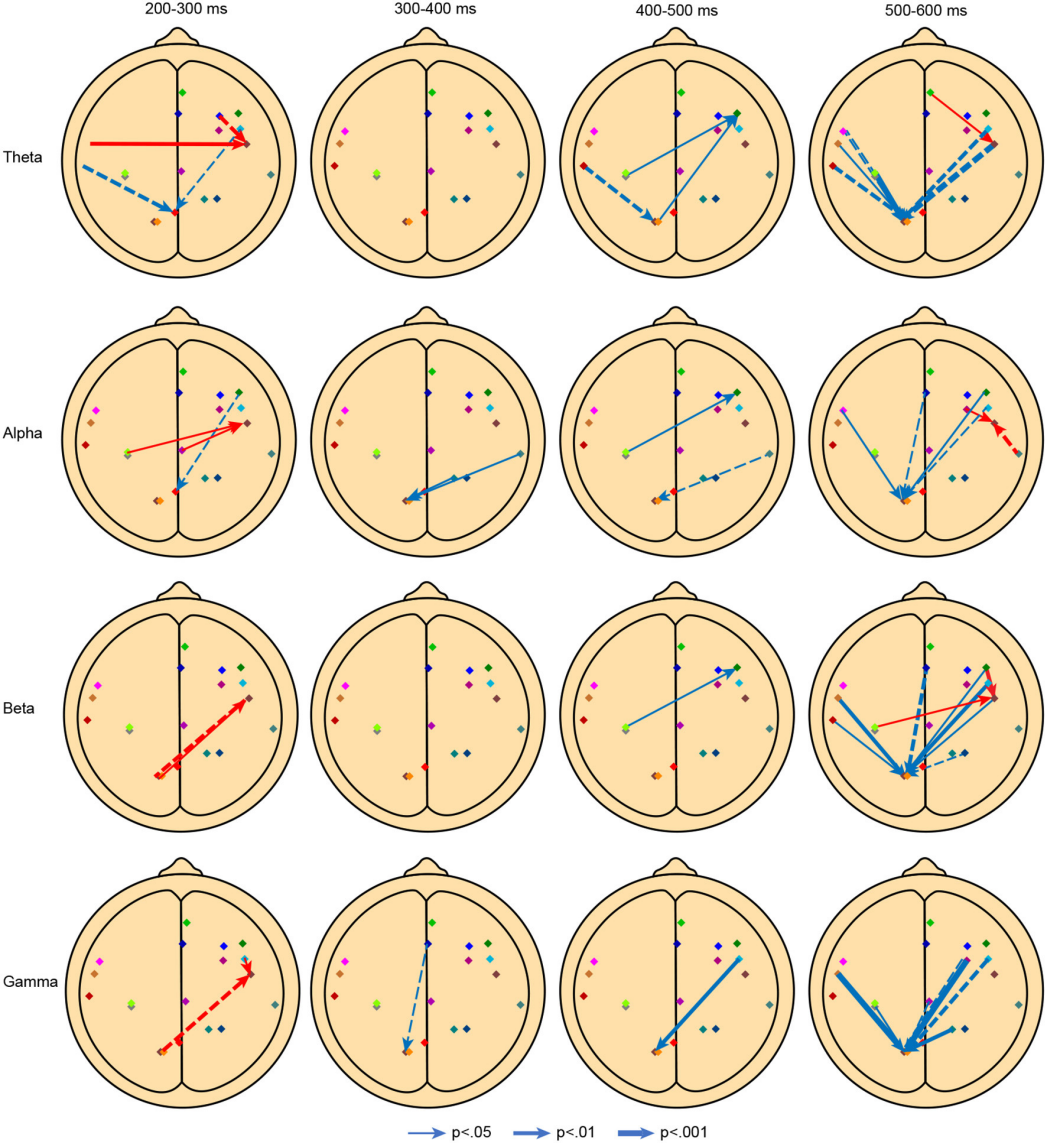
Significant differences in the PDCs among the nineteen ROIs in four time windows and four frequency bands. The arrows indicate the direction of the information flow, and the widths indicate the significance level. The red arrows indicate positive differences in the PDC (perspective-shift and no-perspective-shift conditions), whereas the blue arrows indicate negative differences in the PDC between the two conditions. The dotted lines indicate connectivities that were significantly correlated with the individual characteristics of the participants, which are presented in more detail in Table 4.

We calculated the mean PDCs for the perspective-shift and no-perspective-shift conditions for each participant in the θ, α, β, and γ bands for every 100-ms time window from 200 to 600 ms, for which we observed significant β suppression on the scalp for the perspective-shift condition compared with the no-perspective-shift condition. The PDC values of the two conditions for 171 pairs between the 19 ROIs in two directions (342 PDC values in total) for each time window and the frequency bands were compared using paired-sample *t* tests with corrections for multiple comparisons using non-parametric cluster permutation testing (*N* = 1,000 permutations) (Maris and Oostenveld, 2007). Table 3 shows the destinations and sources of significantly increased and decreased information flow evaluated by the PDC as an effect of the perspective-shift vs. no-perspective-shift condition for three processing phases, the nineteen ROIs, and different frequency bands. Figure 4 schematically shows the significant differences in the PDCs between the perspective-shift and no-perspective-shift conditions for the nineteen ROIs in four time windows and four frequency bands.

**Table 3:** Destinations and sources of significantly increased and decreased information flow (IF) evaluated by the PDC as an effect of the perspective-shift vs. no-perspective-shift condition, for three processing phases, nineteen ROIs, and different frequency bands (FBs).

**200–300 ms**	**ROI, FB**	**300–400 ms**	**ROI, FB**	**500–600 ms**	**ROI, FB**
**•IF increase to r_STC (R17)**		**•IF decrease to precuneus (R1, R13)**		**•IF increase to r_STC (R17)**	
**r_anterior insula** ^**^	R2, θ	**dmPFC** ^*^	R8, γ	vmPFC^*^	R3, θ
l_precentral gyrus^**^	R5, θ	r_SPC^*^	R14, α	r_MFG^*^	R9, α
r_dlPFC^*^	R6, γ	r_STC^*^	R16, α	r_dlPFC^**^	R10, β
l_SMC^*^	R11, α			l_SMC^*^	R11, β
r_MCC/precuneus^*^	R12, α	**400–500 ms**		**r_STC** ^**^	R16, α
**l_PCC/precuneus** ^**^	R13, β, γ	•**IF decrease to r_dlPFC (R10)**		**•IF decrease to precuneus (R13)**	
l_MOG^*^	R18, β	l_SMC^*^	R11, θ, α, β	**l_IFG** ^*^	R4, θ, α
**•IF decrease to precuneus (R1)**		l_PCC/precuneus^*^	R13, θ	l_precentral gyrus^**^/^***^	R5, β/γ
**l_STG** ^**^	R7, θ	**•IF decrease to precuneus (R13)**		**r_dlPFC** ^*^ **/** ^**^	R6, α/θ, β, γ
**r_dlPFC** ^*^	R10, θ, α	**r_dlPFC** ^**^	R6, γ	**l_STG** ^**^	R7, θ
		**l_STG** ^**^	R7, θ	**dmPFC** ^*^ **/** ^**^	R8, α/β
		**r_STC** ^*^	R16, α	**r_MFG** ^*^	R9, γ
		**l_MOC** ^*^	R18, β	r_dlPFC^*^	R10, α
				l_SMC^*^	R11, γ
				r_SPC^**^	R14, γ
				**r_Hippocampus gyrus** ^*^	R15, β
				**r_STC** ^***^ **/** ^*^	R17, θ/β
				**l_MOC** ^*^	R18, β
				l_insula^***^	R19, θ
				**•IF decrease to l_MOC (R18)**	
				**l_IFG** ^*^	R4, θ
				l_precentral gyrus^*^	R5, θ
				l_STG^*^	R7, β
				r_MFG^***^	R9, γ

* *p* < 0.05,

** *p* < 0.01,

*** *p* < 0.001. r_, right; l_, left; SPC, superior parietal cortex; MFG, middle frontal gyrus; STG, superior temporal gyrus; MOC, middle occipital cortex; MCG, middle occipital gyrus. “•” indicates the increased or decreased information flow and the destination (ROI), which is followed by the list of the sources (ROIs and frequency bands). The bolded sources indicate the connectivities for which the individual properties of the participants were significant in the multiple regression analyses, with the difference in the PDC between the no-perspective-shift and perspective-shift conditions as the dependent variable and with the participants' AQ subscales, sex, and age as independent variables (see Table 4 for the details).

#### 3.3.4. Individual differences in effective connectivity

Table 4 shows the variations in the significant effective connectivities and their structures for four time windows and four frequency bands for which the individual properties of the participants were significant in the multiple regression analyses, with the difference in the PDC between the no-perspective-shift and perspective-shift conditions as the dependent variable and with the participants' AQ subscales, sex (female as 0 and male as 1), and age as independent variables.

**Table 4:** Effective connectivities and their structures for four time windows and four frequency bands for which the individual properties of the participants were significant in multiple regression analyses, with the difference in the PDC between the no-perspective-shift and perspective-shift conditions as the dependent variable and with the participants' AQ subscales, sex (female as 0 and male as 1), and age as independent variables.

**200–300 ms**	**Effect**	**300–400 ms**	**Effect**	**500–600 ms**	**Effect**
IF increase to right STC (R17)		**IF decrease to precuneus (R13)**		IF increase to r_STC (R17)	
•r_anterior insula (R2, θ)	3.68^**^	• dmPFC (R8, γ)	−2.98^*^	•r_STC (R16, α)	3.44^**^
Age	0.70^**^	Local Details	0.49^*^	Social skill	−0.48^*^
Communication	0.60^*^	Male	−0.45^*^	**IF decrease to precuneus (R13)**	
Social skill	−0.60^*^			• l_IFG (R4, θ)	−3.17^**^
•l_PCC/precuneus (R13, β)	3.40^**^	**400–500 ms**		Imagination	0.80^***^
Attention switching	−0.48^*^	**IF decrease to precuneus (R13)**		Attention switching	−0.75^**^
– (γ)	3.21^**^	• r_dlPFC (R6, γ)	−3.73^**^	Local Details	0.63^***^
Male	0.50^*^	Local details	0.51^*^	Age	−0.49^*^
Local details	0.41^*^	Age	0.42^*^	• r_dlPFC (R6, θ)	−3.74^**^
Age	0.40^*^	• l_STG (R7, θ)	−3.94^**^	Local details	0.66^***^
**IF decrease to precuneus (R1)**		Local details	0.53^*^	Male	−0.39^*^
• l_STG (R7, θ)	−3.76^**^	Attention switching	−0.41^*^	– (α)	−3.09^*^
Age	0.43^*^	• r_STC (R16, α)	−3.43^*^	Local Details	0.43^*^
• r_dlPFC (R10, θ)	−3.12^*^	Attention switching	−0.55^**^	– (γ)	−4.09^**^
Imagination	−0.66^**^	• l_MOC (R18, β)	−3.04^*^	Local details	0.64^**^
– (α)	−2.98^*^	Male	−0.55^**^	Male	−0.40^*^
Imagination	−0.53^*^	Local details	0.51^**^	• l_STG (R7, θ)	−3.56^**^
Local details	−0.51^*^			Imagination	0.45^*^
				• dmPFC (R8, α)	−3.16^*^
				Attention switching	−0.70^**^
				Social skill	0.54^*^
				Male	0.47^*^
				– (β)	−3.43^**^
				Attention switching	−0.93^**^
				Communication	0.65^*^
				Male	0.46^*^
				• r_MFG (R9, γ)	−3.07^*^
				Attention switching	−0.94^**^
				Imagination	0.75^**^
				Age	−0.52^*^
				• r_Hippocampus gyrus (R15, β)	−3.27^*^
				Attention switching	−0.43^*^
				• r_STC (R17, θ)	−4.03^***^
				Local details	0.59^**^
				• l_MOC (R18, β)	−3.19^*^
				Attention switching	−0.81^**^
				Communication	0.65^*^
				**IF decrease to l_MOC (R18)**	
				• l_IFG (R4, θ)	−3.23^*^
				Age	−0.48^*^

r_, right; l_, left.

* *p* < 0.05,

** *p* < 0.01,

*** *p* < 0.001. The connectivities for which the difference was positive are underlined. The individual properties that were significantly correlated with the PDC difference are listed with their standard partial regression coecients in order of their absolute value (“–” indicates the same structure).

## 4. Discussion

### 4.1. Time course of effective connectivity

The first research question considered in this study is whether the processing mechanism associated with perspective taking changes over time during real-time speech processing. We recognized three processing phases on the basis of the direction and increase or decrease in information flow and the ROI acting as the hub, that is, the time window of 200–300 ms was the first phase, that of 300–500 ms was the second, and that of 400–500 ms was the third. We discuss the properties of the three phases below.

#### 4.1.1. First phase: 200–300 ms

We recognize two hubs in the information flow for the first phase: the right superior temporal cortex (STC, R17) and the precuneus (R1). The flow to the right STC increased, while compared with the flows in the no-perspective-shift condition, the flow to the precuneus decreased in the perspective-shift condition.

One of the hubs, the right STC (R17), is part of both the extended mentalizing network (Overwalle, 2009; Mar, 2011) and the mirror neuron network (Iacoboni et al., 2001). According to the functional connectivity analysis in Hashimoto et al. (2016), the difference in the main effect of perspective between TD and ASD speakers was greatest in the right STC. The sources for which the information flow to the right STC (R17) increased were the right anterior insula (R2), left precentral gyrus (R5), right dlPFC (R6), left SMC (R11), right MCC/precuneus (R12), left PCC/precuneus (R13), and left middle occipital cortex (R18). According to Komeda et al. (2015), The functional connectivity associated with evaluating nonautistic characteristics was significantly greater for TD speakers than for ASD speakers in the left precentral gyrus and the right dlPFC, where the vmPFC was taken as the seed. The dlPFC is the main node in the executive control network (Bauer et al., 2020), and recent studies have suggested that it is part of the mentalizing network and is linked to cognitive empathy (Abu-Akel and Shamay-Tsoory, 2011; Isernia et al., 2021). The precuneus is part of the mentalizing network and is the functional core of the default mode network (Utevsky et al., 2014, 2016; Yeshurun et al., 2021). According to the analysis in Hashimoto et al. (2016), the main effect of perspective was significantly greater in the left middle occipital cortex for TD speakers than for ASD speakers.

The sources for which the information flow to the precuneus, the other hub in the first phase, decreased were the left STG (R7) and right dlPFC. The connectivity between the precuneus and the left STG can be understood as an interaction between the mentalizing network and the language processing network. Reportedly, the time course of neural responses in a speaker's left STG is correlated with that of the listener in a dialog (Dikker et al., 2014), which suggests that the left STG is associated with the interactions between speakers in real-time speech communication.

The first phase is characterized mainly by interactions in the mentalizing network and by increased information flow from the mirror neuron network and the executive control network to the mentalizing network. Moreover, we note the beginning of the function of the precuneus as a functional core, which was salient in the third phase.

#### 4.1.2. Second phase: 300–500 ms

In the first half (300–400 ms) of the second phase, the precuneus (R1, R13) functioned as a hub. The sources for which the information flow to the precuneus decreased were the dmPFC (R8), right superior parietal cortex (R14), and right STC (R16). The dmPFC was the seed in Komeda et al. (2015), and it is the main area for making judgments on others (D'Argembeau et al., 2007). The meta-analysis by Bzdok et al. (2013) demonstrated that the dmPFC is selectively associated with perspective taking and the retrieval of autobiographical memory. The right superior parietal cortex is part of the mirror neuron network (Rizzolatti and Craighero, 2004; Brass and Heyes, 2005), and according to the connectivity analysis in Hashimoto et al. (2016), the difference in the main effect of perspective was second greatest between TD and ASD speakers in this region. Hashimoto et al. (2016) suggested that self-evaluations and other evaluations from the third-person perspective require memory retrieval and reasoning, which activate the attentional system in the right parietal cortex (Legrand and Ruby, 2009).

The hubs in the latter half (400–500 ms) of the second phase were the right dlPFC and precuneus. The sources for which the information flow to the right dlPFC decreased were the left SMC and precuneus. According to the meta-analysis of Schomers and Pulvermüller (2016), the left SMC represents the phonological information in speech perception. Here, we can recognize the importance of the executive control network in speech processing.

The sources for which the information flow to the precuneus decreased were the right dlPFC, left STG, right STC (R16), and left middle occipital cortex. The second phase is characterized by changes in connectivity from the dmPFC to the precuneus and changes in connectivity from the left SMC to the right dlPFC, which were observed in several frequency bands, including θ, α, and β. We can recognize a significant interaction between the mirror neuron network and executive control network.

#### 4.1.3. Third phase: 500–600 ms

The hubs in the third phase were the right STC, left PCC/precuneus, and left middle occipital cortex. The information flow to the right STC (R17) increased, whereas the flow to the left PCC/precuneus and left middle occipital cortex decreased.

The sources for which the information flow to the right STC (R17) increased were the vmPFC, right MFG (R9), right dlPFC, and left SMC. Increased information flow to the right STC (R17) was also observed in the first phase, but significantly increased flow from the vmPFC and right MFG was only recognized in the third phase.

In the third phase, the precuneus receives information from almost all the ROIs, and we recognize the importance of the precuneus as the functional core. In particular, the decreased information flow from the right dlPFC to the precuneus was significant in all frequency bands, and we note the importance of the executive control network in perspective shifting.

The third hub is the left middle occipital cortex. Its sources were the left IFG (R4), left precentral gyrus, left STC, and right MFG, and they were subsets of the sources of the flow to the precuneus.

As evidenced by the mix of PDC increases and decreases throughout the three phases, perspective taking cannot be understood solely in terms of increased effective connectivity. The destination with increased information flow is the right STC, and the destinations with decreased information flow are the right dlPFC and precuneus. Here, we can emphasize the interactions between the executive control network and the mentalizing network as well as the function of the precuneus as the functional core. To our knowledge, this is the first study to report temporal variations in the effective connectivity associated with perspective taking in real-time speech comprehension.

### 4.2. Relevance of time frequency bands

The second research question of this study is whether the effective connectivity associated with perspective taking differs across frequency bands. We observed significant β suppression for perspective shift on the scalp, but the effective connectivity at the source level widely varied from the θ to γ bands. However, we also note that the connectivity associated with the language areas was significant mainly in the θ band. The left STG was the source of significant connectivity to the precuneus and left PCC/precuneus in the first, second, and third phases in the θ band (although the connectivity from the left STG to the left MOC in the third phase was significant in the β band). Similarly, the connectivities from the left IFG to the precuneus and the left MOC were significant in the θ band, although the former connectivity was also significant in the α band.

Linguistic ERP is often characterized by the θ band. Schneider et al. (2016) reported that adult speakers showed widely distributed θ and β suppression when identifying grammatical errors. Furthermore, Schneider and Maguire (2018) examined the relationship between ERPs and temporal frequency through syntactic and semantic anomalies in English. Schneider and Maguire (2018) demonstrated that N400 was associated with increased power in the θ band, whereas P600 was associated with suppression in the β band. For the control sentences in the present study, negative and positive ERPs were observed in the frontal and parietal regions, respectively. Their signal intensity was suppressed in the β band, and the intensity of the frontal negativity was also suppressed in the θ band (Tokimoto and Tokimoto, 2018). These findings suggest a close relationship between language processing and the θ band, and the present study suggests that the θ band encoded significant linguistic information in brain connectivity networks.

### 4.3. Individual differences in sociality

The third research question is whether the effective connectivity associated with perspective taking varies with individual differences in sociality, including sex and age, among typically developing individuals. We note the special role of attention switching in the correlation between individual differences in sociality and effective connectivity. As shown in Table 4, in the multiple regression analyses with the change in PDC due to perspective shifts as the dependent variable and with the AQ subscales, sex, and age as independent variables, the standardized partial regression coefficients for attention switching were all negative. However, the significant effects of the other AQ subscales were either positive or negative depending on the processing phase and on the increase or decrease in information flow.

In the connectivities in the first phase, the sign of the standardized partial regression coefficient of the subscale was almost always consistent with the increase or decrease in the PDC. That is, when the information flow increased, the coefficient of the subscale was positive, and when the flow decreased, the coefficient was negative. However, for the flow increase from the left PCC/precuneus to the right STC with the significant effect of attention switching, its coefficient was negative.

In the second phase (400–500 ms), the two standardized partial regression coefficients of attention switching for the decreased information flow were negative, whereas the four coefficients of local details were positive.

Similarly, for the decreased information flow in the third phase (500–600 ms), the six significant coefficients of attention switching were all negative again, whereas the coefficients of the other subscales were negative for increased flow and positive for decreased flow. Thus, for the connectivities in the second and third phases, the signs of the coefficients of the subscales other than attention switching were opposite to the sign of the PDC change. Table 5 summarizes the correspondences between the PDC effect in the perspective-shift condition, the standardized partial regression coefficients of attention switching and the other AQ subscales, and the connectivity direction for the right dlPFC for the three time windows. This tendency suggests that attention switching plays a special role in shifting perspective during speech comprehension.

**Table 5:** Correspondences between the PDC effect for the perspective-shift condition, the standardized partial regression coefficients of attention switching and the other AQ subscales, and the connectivity direction for the right dlPFC for the three time windows.

**Time window**	**PDC effect**	**β of attention switching**	**β of the other subscales**	**Right dlPFC**
200–300 ms	Positive	Negative	Positive^*^	Source
	Negative		Negative	
400–500 ms	Negative	Negative	Positive	Destination
500–600 ms	Positive		Negative	
	Negative	Negative	Positive	Source

* The coefficient of social skills for the PDC increase from the right anterior insula (R2, theta) to the right STC (R17) was exceptionally negative.

We also note the different roles of the right dlPFC in the various time windows. In the time window of 200–300 ms, the right dlPFC was the source of information flow, whereas in the window of 400–500 ms, it was the destination of the flow. Furthermore, in the window of 500–600 ms, the right dlPFC was the source of information flow again. This result indicates a significant change in the involvement of the executive control network in the three phases. These systematic changes suggest that perspective taking consisted of three phases with respect to the involvement of the executive control network. We can also recognize that the precuneus was always involved in connectivity where individual differences in attention switching were significant.

Regarding the effect of sex, we found seven connectivities for which the effect of sex was significant, and the precuneus was the destination for six of the seven and the source for the remaining connectivity. Thus, we emphasize the important role of the precuneus in the differences between males and females. Furthermore, in four of the five connectivities (seven when frequency band is considered) where sex differences are significant, the coefficient of male is consistent with an increase or decrease in PDC. This study confirmed the effect of sex in the process of perspective taking as an operation of the theory of mind in real time. However, the effect of sex is not consistent for phase, connectivity region or frequency band. It is thus difficult to generalize conclusions at present.

Regarding the effect of age, the participants in this study were aged from 20 to 46, which corresponds to a phase of gradual decline of empathy, according to Greenberg et al. (2023). In this study, the standardized partial regression coefficient of age is positive in the first and second phases but negative in the third phase. Thus, age is associated with multiple processing phases. For the correspondence between the increase or decrease in PDC due to perspective shift and the standardized partial regression coefficient of age, the signs are consistent for five of the seven significant connectivities. However, we do not see the correspondence as a very strong trend, and it is difficult to generalize conclusions at present.

To our knowledge, the present study is the first to show that individual differences in sociality, including sex and age differences, are systematically associated with the neural network for perspective taking as a fundamental communication ability in typically developing speakers.

## 5. Concluding remarks

The present study showed that perspective taking in speech comprehension is realized by interactions among the mentalizing network, mirror neuron network, and executive control network and that this processing involves multiple stages. Furthermore, we found that individual differences in sociality, including sex and age, were systematically associated with effective connectivity and that switching attention played an important role in perspective taking. In particular, precuneus, as a functional core, played a special role in implementing individual differences.

## 6. Limitations and future directions

Finally, let us enumerate the remaining problems in this study and future directions.

We have shown the interactions for perspective taking between the mentalizing network, mirror neuron network, and executive control network with the direction of information flow and their temporal change in real-time speech comprehension. Furthermore, we have demonstrated that individual differences in sociality, including sex and age, are systematically associated with the interaction between the networks. We have also noted that attention switching plays an important role in perspective taking and that the precuneus plays a crucial role in implementing individual differences. However, the exact function of connectivity between individual ROIs is unknown. Furthermore, as briefly noted in the discussion section, changes in effective connectivity were both increasing and decreasing, and we must discuss the positive significance of a decrease in information flow. Furthermore, consistent theoretical generalizations have not been obtained regarding the effect of sex and age on effective connectivity. Since the understanding of effective connectivity in language processing is still insufficient, new experimental findings on effective connectivity in other kinds of language processing are necessary. In particular, Tokimoto and Tokimoto (2018) found common neural activity between perspective taking in facial expression recognition and sentence comprehension, suggesting that the process of perspective taking is modality-general. Therefore, to understand the exact function of effective connectivity in language processing, new studies on the effective connectivity not only in language processing but also in other types of cognitive processing, including face recognition in different modalities, will be instructive. Furthermore, in addition to accumulating findings on effective connectivity in language and cognitive processing, a theoretical framework on brain-wide connectivity is needed to accurately interpret the function of individual connections and provide new research perspectives.

It is appropriate to point out here for future research that some Japanese verbs other than *ageru* and *kureru* can also behave as subsidiary verbs to indicate a perspective other than that of the speaker. One of them is *morau* (receive), which requires the perspective of the subject. In (11-a), for example, *-moratta* (received) indicates that the event is described from the perspective of the subject, *watashi* (I, the speaker). The anomaly of (11-b) can be understood as a violation of the ban on conflicting linguistic empathy foci in (5). That is, two different perspectives are present in the sentence, namely, the perspective of the subject, *Maruyama-san* (Mr./Ms. Maruyama), required by *-moratta*, and that required by *watashi* (I). In (11-c), therefore, the perspective is expected to shift from the speaker as the default to *Matsunaga-san* (Mr./Ms. Matsunaga) at *-moratta*. The subsidiary verbs and the corresponding foci of the perspectives are underlined here.

(11)    a.    Watashi-ga  Maruyama-san-ni          bentoo-o
I-nom         Mr./Ms. Maruyama-dat lunch box-acc
katte-moratta.
buy-received
“I had Mr./Ms. Maruyama buy a lunch box (for me).”
    b.    *Maruyama-san-ga          watashi-ni bentoo-o
Mr./Ms. Maruyama-nom I-dat          lunch box-acc
katte-moratta.
buy-received (for him/her)
“Mr./Ms. Maruyama had me buy a lunch box (for
him/her).”
    c.    *Matsunaga-san-ga           Maruyama-san-ni
Mr./Ms. Matsunaga-nom Mr./Ms. Maruyama-dat
bentoo-o          katte-moratta.
lunch box-acc buy-received
“Mr./Ms. Matsunaga had Mr./Ms. Maruyama buy a
lunch box (for Mr./Ms. Matsunaga).”

*Iku* (go) and *kuru* (come) can also function as subsidiary verbs to indicate a perspective other than that of the speaker. In (12-a), for example, *-itta* (past form of *iku*) indicates that the speaker takes a perspective close to (that of a person at) the departure point, that is, the location of the subject, *watashi*. The example in (12-b) is awkward because *-itta* requires a perspective close to that of *Maruyama-san* (Mr./Ms. Maruyama), whereas the presence of *watashi* requires the perspective of the speaker. In (12-c), therefore, the perspective is expected to shift from the speaker to *Matsunaga-san* (Mr./Ms. Matsunaga) at *-itta*.

(12)    a.    Watashi-ga  Maruyama-san-ni  bentoo-o  katte-itta
(buy-went).
“I bought a lunch box and went to him/her.”
    b.    *Maruyama-san-ga  watashi-ni  bentoo-o  katte-itta
(buy-went).
“Mr./Ms. Maruyama bought a lunch box and went to
me.”
    c.    Matsunaga-san-ga Maruyama-san-ni bentoo-o katte-
itta (buy-went).
“Mr./Ms. Matsunaga bought a lunch box and went to
Mr./Ms. Maruyama.”

On the other hand, *-kuru* (come) indicates that the speaker takes a perspective close to the arrival point. The example in (13-a) with *-kita* (past form of *-kuru*) is described from the perspective of (a person at) the arrival point, namely, the location of the dative object (the speaker). We can find an interesting property of *kuru* concerning perspective-taking in (13-b). The example in (13-b) can theoretically be considered anomalous because two different perspectives are present, namely, the perspective of *Maruyama-san* (Mr./Ms. Maruyama), required by *-kita*, and the perspective of the speaker, required by *watashi*. However, (13-b) is not especially awkward, probably because the comprehender can retrieve the context in which both the speaker and *Maruyama-san* are at the same point. In this context, the perspective required by *-kita* and that required by *watashi* are consistent with each other. We can here predict some measurable cost of retrieving the appropriate context for the interpretation of (13-b). In (13-c), the perspective is expected to shift from the speaker to Maruyama-san (Mr./Ms. Maruyama) at *-kita* in the same way as at *-itta*.

(13)    a.    Maruyama-san-ga  watashi-ni  bentoo-o  katte-kita
(buy-came).
“Mr./Ms. Maruyama bought a lunch box and came to
me.”
    b.    Watashi-ga  Maruyama-san-ni  bentoo-o  katte-kita
(buy-came).
“I bought a lunch box and came to him/her.”
    c.    Matsunaga-san-ga Maruyama-san-ni bentoo-o katte-
kita (buy-came).
“Mr./Ms. Matsunaga bought a lunch box and went to
Mr./Ms. Maruyama.”

*Ageru* also has a colloquial form, *yaru* (give), as exemplified in (14-a). Furthermore, *ageru* and *kureru* have honorific forms, namely, *sashiageru* in (14-b) and *kudasaru* in (14-c), respectively.

(14)    a.    Watashi-ga Maruyama-san-ni          bentoo-o
I-nom        Mr./Ms. Maruyama-dat lunch box-acc
katte-yatta.
buy-gave (to him/her)
“I bought a lunch box for Mr./Ms. Maruyama.”
    b.    Watashi-ga sensei-ni     bentoo-o
I-nom        teacher-dat lunch box-acc
katte-sashiageta.
buy-gave (honorific to the dative object)
“I bought a lunch box for my teacher.”
    c.    Sensei-ga      watashi-ni bentoo-o
teacher-nom I-dat         lunch box-acc
katte-kudasatta.
buy-gave (honorific to the subject)
“My    teacher    bought    a    lunch
box for me.”

Therefore, we can examine the interaction between the perspective shift and the relative social power by manipulating *yaru, sashiageru*, and *kudasaru* with *ageru* and *kureru* as the controls.

In this study, the stimulus utterances were made by a female voice to prioritize the accuracy of speech recognition, but different neural activity could appear if the experimental voice were male, since the effect of sex was significant in several connectivities. Possible interactions between the sex of the stimulus voice and that of the participants are a topic for future study.

## Data availability statement

The raw data supporting the conclusions of this article will be made available by the authors, without undue reservation.

## Ethics statement

The studies involving humans were approved by Ethics Committee of Shobi University. The studies were conducted in accordance with the local legislation and institutional requirements. The participants provided their written informed consent to participate in this study.

## Author contributions

ST: study design, data acquisition, data analysis, data interpretation, and manuscript preparation. NT: data acquisition, data analysis, and data interpretation. All authors contributed to the article and approved the submitted version.

## Footnotes

^1^Most of the data (21 participants) were borrowed from Tokimoto and Tokimoto (2018), who analyzed the relationship between the neural substrates associated with perspective taking and temporal processing. The present study is a new analysis of the effective connectivity associated with perspective taking during real-time utterance comprehension.

^2^The left superior temporal gyrus [(−68, −16, 0) in MNI coordinates] is discussed in the functional connectivity analysis performed in Hashimoto et al. (2016). This region is essentially the same as R7 in Table 2, which was selected according to Komeda et al. (2015), and therefore, the two regions are united as R7 in this study.
